# Diagnostic Value of Vestibular Evoked Myogenic Potentials in Endolymphatic Hydrops: A Meta-Analysis

**DOI:** 10.1038/srep14951

**Published:** 2015-10-12

**Authors:** Sulin Zhang, Yangming Leng, Bo Liu, Hao Shi, Meixia Lu, Weijia Kong

**Affiliations:** 1 Department of Otorhinolaryngology, Union Hospital, Tongji Medical College, Huazhong University of Science and Technology, Wuhan, Hubei, China; 2 Institute of Otorhinolaryngology, Union Hospital, Tongji Medical College, Huazhong University of Science and Technology, Wuhan, Hubei, China; 3 Key Laboratory of Neurological Disorders of Education Ministry, Tongji Medical College, Huazhong University of Science and Technology, Wuhan, Hubei, China; 4 Department of Epidemiology and Biostatistics, and the Ministry of Education Key Lab of Environment and Health, School of Public Health, Tongji Medical College, Huazhong University of Science and Technology, Wuhan, Hubei, China

## Abstract

In this study, we evaluated the clinical diagnostic value of vestibular evoked myogenic potentials (VEMPs) for endolymphatic hydrops (EH) by systematic review and Meta-analysis. The pooled sensitivity, specificity, positive likelihood ratio, negative likelihood ratio, diagnostic odds ratio and area under summary receiver operating characteristic curves (AUC) were calculated. Subgroup analysis and publication bias assessment were also conducted. The pooled sensitivity and the specificity were 49% (95% CI: 46% to 51%) and 95% (95% CI: 94% to 96%), respectively. The pooled positive likelihood ratio was 18.01 (95% CI: 9.45 to 34.29) and the pooled negative likelihood ratio was 0.54 (95% CI: 0.47 to 0.61). AUC was 0.78 and the pooled diagnostic odds ratio of VEMPs was 39.89 (95% CI: 20.13 to 79.03). In conclusion, our present meta-analysis has demonstrated that VEMPs test alone is not sufficient for Meniere’s disease or delayed endolymphatic hydrops diagnosis, but that it might be an important component of a test battery for diagnosing Meniere’s disease or delayed endolymphatic hydrops. Moreover, VEMPs, due to its high specificity and non-invasive nature, might be used as a screening tool for EH.

Meniere’s disease (MD) is a well-known inner ear disorder. Its symptoms include recurrent episodes of self-limiting vertigo, fluctuating or progressive sensorineural hearing loss, fullness and tinnitus of the affected ear. Previous studies showed that endolymphatic hydrops (EH) is a major histopathological characteristics of MD. Over the past two decades, mounting evidence has demonstrated that MD may present a variety of clinical symptoms and respond differently to treatment and possesses a wide array of phenotypical and endophenotypical features of inner ear disorders^1^,^2^,^3^.

Delayed endolymphatic hydrops (DEH) is defined as delayed development of episodic vertigo following either ipsilateral or contralateral ear with profound sensorineural hearing loss^4^.

Underlying pathological state of MD is idiopathic EH, while, DEH is one form of secondary EH. Therefore, pathophysiologically, both DEH and MD have EH^1^,^2^,^3^,^4^. Previous studies suggested that development of EH involves a set of environmental, genetic and epigenetic factors. However, the underlying pathogenesis of EH remains poorly understood. Up to date, no single method, neither physical examinations nor diagnostic tests, can identify EH with significant certainty^5^.

Currently, MD and DEH are principally diagnosed on the basis of the typical clinical symptoms, pure tone audiometry and the presence of EH^1^. However, the objective *in vivo* confirmation of EH is difficult and it might be subject to constant change during a vertigo attack and between attacks. Since vestibular or cochlear symptoms may occur separately at the early stage of EH, clinical diagnosis of EH can be difficult. Previous studies indicated that the remission rate of stood somewhere between 60% to 80% in MD patients receiving treatments^6^. To improve the effectiveness of clinical treatment, it is of great importance to establish a reliable technique for diagnosing EH.

Vestibular evoked myogenic potentials (VEMPs) can be used for assessing the otolith organ and peripheral vestibular function^7^,^8^. Cervical vestibular evoked myogenic potentials (c-VEMPs) can serve as an indicator of vestibular function. c-VEMPs are elicited via a special pathway that goes from the saccule, inferior vestibular nerve, vestibular nucleus, medial, lateral vestibulospinal tract and finally to the ipsilateral sternocleidomastoid muscle (SCM)^9^. Moreover, ocular vestibular evoked myogenic potentials (o-VEMPs), another indicator of vestibular function, are evoked through the pathway that starts from utricle, superior vestibular nerve, vestibular nucleus, the medial longitudinal fasciculustill, oculomotor nuclei and ends at the contralateral extraocular muscles^10^. Distortion of the membranous labyrinth, labyrinthine ruptures, and complete collapse of the membranous labyrinth may disturb the homeostasis of the inner ear. These may explain symptoms in auditory and vestibular systems in EH, the histopathological hallmark of MD and DEH, tends to develop in the cochlea. It then extends to the saccule and utricle and eventually involves the semicircular canals. In fact, anatomic studies of temporal bone suggested that the functional impairment in the cochlea, saccule, utricle, and semicircular canals may be the consequence of sequential development of EH^11^.

Moreover, abnormal VEMPs can also be recorded in other vertigo diseases, such as vestibular neuritis, superior canal dehiscence, benign paroxysmal positional vertigo, sudden haring loss, vestibular Schwannoma, multiple sclerosis, Miller Fisher syndrome and so on^9^,^10^,^11^,^12^. In all these conditions, the saccule or inferior vestibular nerves are involved. Differentiation diagnosis relies on detailed history-taking, physical examinations, a battery of audio-vestibular function tests including pure tone audiometry, VNG, MRI on inner ear and brain and CT scan on temporal bone. In this study, we focused on EH, a histopathological hallmark of both MD and DEH, to explore the diagnostic value of VEMPs.

Since VEMPs can be used for detecting EH, we were led to assume that VEMPs test would be helpful in the diagnosis of EH. VEMP is complementary caloric test, rotation test and pure tone audiometry for EH diagnosis. Previous studies have intensively explored the diagnostic value of VEMPs for MD or DEH, and the findings varied substantially with different researches. In this meta-analysis, we comprehensively summarized the results of prior results with an attempt to precisely evaluate the usefulness of VEMPS in the diagnosis of EH due to MD or DEH.

## Results

### Literature search and eligible studies

**Figure 1:**
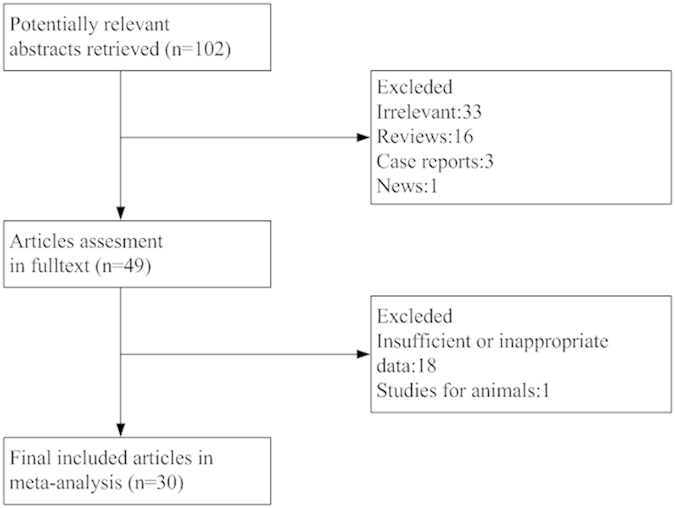
Flowchart for the selection procedure for eligible studies.

Initially, 102 studies were identified after elimination of duplicates (Fig. 1). By screening titles or abstracts against our inclusion/exclusion criteria, 53 articles were excluded (Irrelevant: 33, Reviews: 16, Case reports: 3, News: 1), with 49 full-text articles left. Then another 18 articles were removed for failure to providing sufficient data and 1 animal study was excluded. Our effort to contact the original authors for detailed data failed. In the end, 30 eligible articles were included for our meta-analysis. The features of all eligible studies are presented in Table 1.

### Quality assessment of the included studies

**Figure 2:**
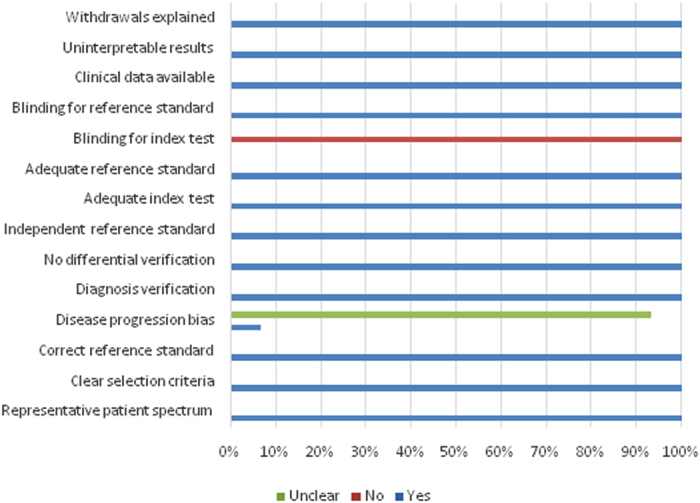
Evaluation of the methodological quality of the included studies according to quality assessment diagnostic accuracy studies tool (QUADAS) criteri.

To evaluate the quality of the eligible studies, we employed the Quality Assessment of Diagnostic Accuracy Studies (QUADAS) tool. The overall quality of the included studies was high, as shown in Fig. 2.

### Meta-analysis

**Figure 3:**
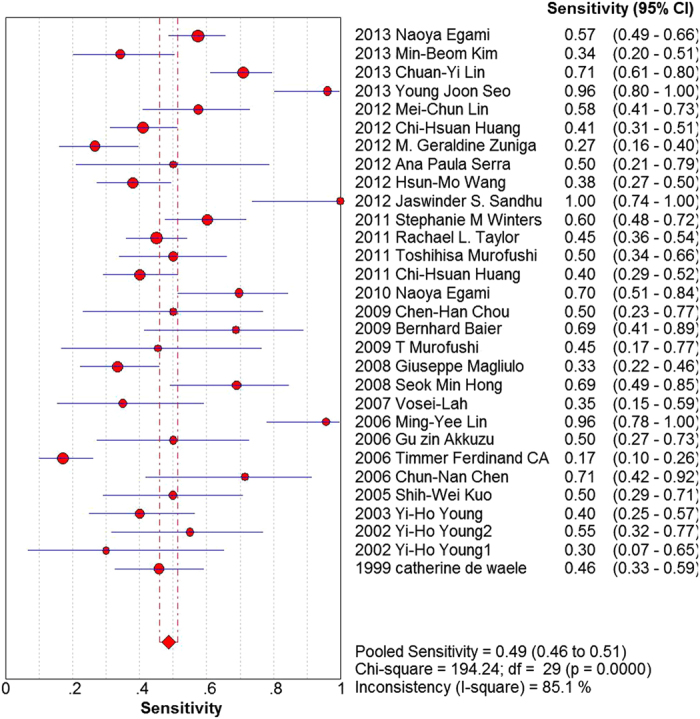
Forest plot of the sensitivity of included studies, summary sensitivity and I^2^ statistic for heterogeneity.

**Figure 4:**
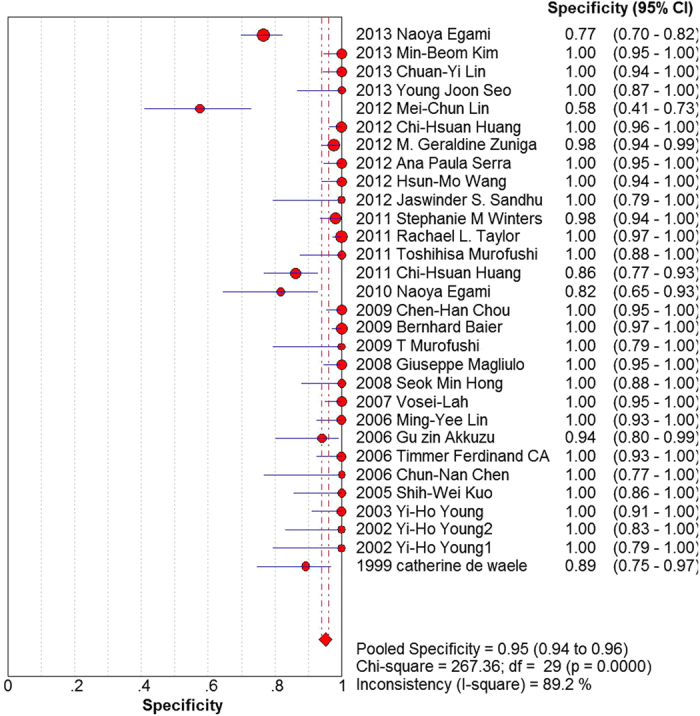
Forest plot of the specificity of included studies, summary specificity and I^2^ statistic for heterogeneity.

**Figure 5:**
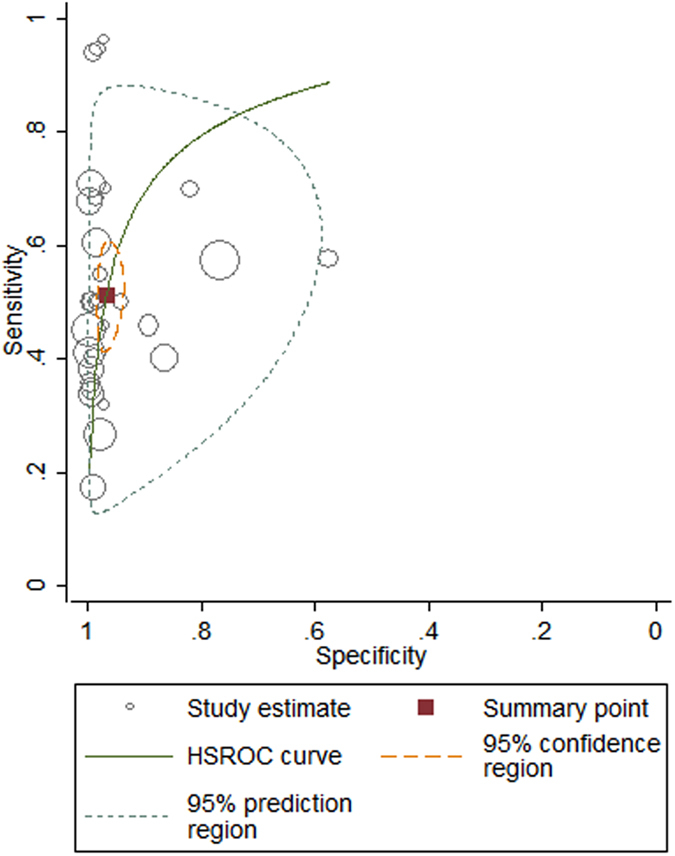
Hierarchical summary receiver operating characteristic curves of VEMPs for detecting EH. The red square represents the summary estimate sensitivity and specificity with 95% confidence region and 95% prediction region for the diagnosis value of EH by VEMPs. The size of the circles indicates the total number in each study.

Our analysis revealed that the pooled sensitivity and the specificity of all studies were 49% (95% CI: 46% to 51%) and 95% (95% CI: 94% to 96%), respectively (Figs 3 and 4). The pooled positive likelihood ratio was 18.01 (95% CI: 9.45 to 34.29) and the pooled negative likelihood ratio was 0.54 (95% CI: 0.47 to 0.61). The area under the summary receiver operating characteristic curve was 0.78 and the pooled diagnostic odds ratio estimate for VEMP was 39.89 (95% CI: 20.13 to 79.03). The SROC graph with the 95% confidence region and with the 95% prediction region is shown in Fig. 5.

### Subgroup Analysis

A subgroup analysis was conducted to identify potential sources of heterogeneity among the included studies (Caucasian patients *vs*. Asian patients, prospective design *vs.* retrospective design, healthy controls *vs.* patient controls, period between attacks *vs.* period during attacks, air conduction *vs.* bone conduction, o-VEMP *vs.* c-VEMP, comparison among different stages, tone burst *vs.* click, and funded projects *vs.* non-funded projects). As shown in Table 2, the sensitivity, specificity and diagnostic accuracy of VEMP test were higher in Caucasian patients, prospective studies, healthy controls, period during attacks, bone conduction, c-VEMP, stage II–IV, tone burst and funded projects, respectively, than in Asian patients, retrospective studies, patient controls, period between attacks, air conduction, o-VEMP, stage I, click and non-funded projects.

### Assessment of Publication Bias

**Figure 6:**
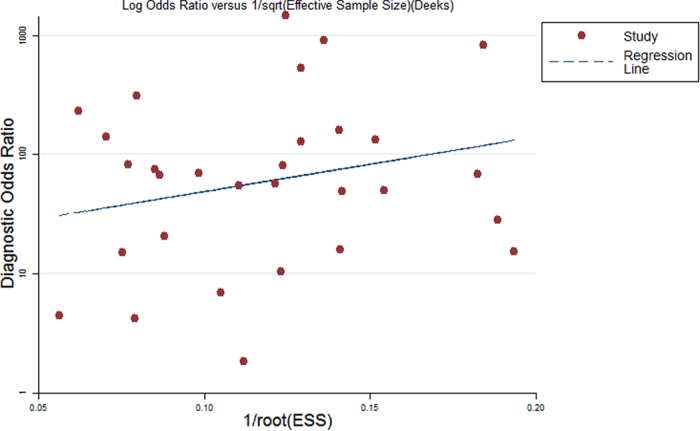
Publication bias was evaluated by Deek’s funnel plots.

To evaluate potential publication bias among the included studies, Deeks’ funnel plot was obtained on the basis of the log diagnostic odd ratios (DOR) and sample size of individual studies. The funnel plot for VEMP is shown in Fig. 6.

## Discussion

Accurately distinguishing between the affected and non-affected sides of EH is crucial for stage assessment, therapeutic planning, disease monitoring and treatment efficacy. In clinical practice, diagnosis of EH remains a great challenge and this is especially true when the auditory symptoms are independent of vestibular ones. Hence, it is of great significance to find a test battery that provides comprehensive assessment of clinical conditions. Conventional vestibular function test (including caloric test and rotation test) detects horizontal semicircular canal involvement while audiometry measures cochlear involvement. Different from these two techniques, VEMPs test accesses the involvement of saccule and utricule. Recently, VEMPs have emerged as a non-invasive approach for diagnosing EH due to MD or DEH^7^,^8^,^9^,^10^.

Since VEMPs are not affected by ipsilateral hearing impairment, some researchers believed that it might be valuable tool for diagnosing, staging and even predicting EH.

Our meta-analysis showed that the sensitivity of VEMPs test in EH patients was 49%. A previous study exhibited that VEMPS had moderate sensitivity and relatively higher specificity as compared with the conventional vestibular function test^13^.

Hence, VEMPs might serve as a useful diagnostic tool for EH. The diagnostic odds ratio (DOR) is an accurate measure, which integrates both sensitivity and specificity. A DOR of 1.0 shows that the test does not distinguish between patients and healthy individuals. In this meta-analysis, the DOR value was 39.89 (95% CI: 20.13 to 79.03), indicating that the accuracy was significant. Moreover, the area under the SROC curve reflects the overall performance of the test for assessing the trade-off between sensitivity and specificity.

Our subgroup analysis demonstrated that the sensitivity, specificity and diagnostic accuracy of VEMP test were higher in Caucasian patients, prospective studies, healthy controls, period during attacks, bone conduction, c-VEMP, stage II–IV, tone burst and funded projects, respectively, than in Asian patients, retrospective studies, patient controls, period between attacks, air conduction, o-VEMP, stage I, click and non-funded projects.

Manzari *et al.*^14^ measured cervical and ocular VEMPs in MD patients during an acute attack and between attacks. Their data illustrated that the signals of both cervical and ocular VEMPs were higher during the vertigo attack than between the attacks. c-VEMP and oVEMP can be applied for assessing the function of different otolith organs. Therefore, the patients with both utricular and saccular disorders tend to have abnormal c-VEMP and o-VEMP amplitudes. Furthermore, for patients with conductive hearing loss, despite presence of middle ear dysfunction, the bone-conducted stimuli are transmitted to the vestibular organs directly through the skull bones. VEMPs of tone burst stimuli are more sensitive and stable than those of click stimuli.

Then, we compared the VEMPs data of various MD stages and found that the diagnostic sensitivity increased with the progression of MD. However, this stage system for MD cannot be applied to DEH cases, since the latter represents as profound sensorineural hearing loss on the lesion ear. In this meta analysis, only one research with DEH stage information was included. With the conventional staging system, the status of MD is assessed only by the means of hearing measurement. In some cases of late stage MD, vestibular function is intact^1^,^2^,^3^ while with the progression of MD, the vestibular function and hearing level fluctuate. Some patients with minimal hearing loss may have sustained saccular hydrops. On the other hand, a patient with maximum hearing loss may have normal saccular function. Hence, clinically, Meniere’s disease falls into two categories: typical MD and atypical one, in terms of cochlear and vestibular symptoms^1^,^2^,^3^. Since the VEMPs test assesses saccular function, the VEMPs may not be correlated with ipsilateral audiometric thresholds^1^,^2^,^3^. The status of the vestibular system can not be audiometrically determined. Therefore, VEMPs may act as a new staging tool in MD diagnosis. It is complementary to the conventional tests such as pure tone audiometry. However, VEMPs result should be interpreted in combination with other clinical features (recurrent episodes, degree of endolymphatic hydrops, timing of detection, disease stages and treatment protocols). Furthermore, a test battery comprising detailed history taking, pure tone audiometry, caloric test, o-VEMP or c-VEMP and image examination may provide an overall assessment of patients’ status.

This study had some limitations. First, the studies included had some heterogeneity. Second, so far, there has been no established gold standard for MD or DEH diagnosis yet. In this meta-analysis, we used clinical history and close follow-up instead as the reference standard. Third, the potential publication bias might exist and Deeks’ funnel plot was employed for evaluating publication bias based on DOR and sample sizes of selected studies. The publication bias among the included studies suggested that the diagnostic value of VEMP on MD identification might be over-estimated, since positive data were prone to being published. Forth, it should be emphasized that these findings were mainly based upon studies of small sample size, and thus further extrapolation should be cautious. Well-designed prospective studies with large patient’s cohorts are warranted to further evaluate the value of VEMP for identifying EH due to MD or DEH.

## Conclusion

In conclusion, our present meta-analysis has demonstrated that VEMPs test alone is not sufficient for MD or DEH diagnosis, but that it might be an important component of a test battery for diagnosing MD or DEH. Moreover, VEMPs, due to its high specificity and non-invasive nature, might be used as a screening tool for endolymphatic hydrops.

## Methods

### Search strategy and study selection

PubMed and Embase were searched to identify eligible studies published before 2015. We used terms “vestibular evoked myogenic potential”, “Meniere’s Disease”, “Endolymphatic hydrops”, “Delayed Endolymphatic hydrops” and “vestibular hypofunction”. In addition, we also manually searched reference lists from related reviews and all retrieved articles. The language was restricted to English. Duplicate publications were removed.

### Selection criteria

Studies included in this meta-analysis met the following criteria:

1. They were about the assessment of the diagnostic accuracy of vestibular evoked myogenic potentials in Meniere’s disease, endolymphatic hydrops or delayed endolymphatic hydrops;
2. They were about the measurement of the sensitivity and specificity of vestibular evoked myogenic potentials.
3. They included detailed case history, follow-up observations and pure tone audiometry as the reference standard. All subjects were identified as either definite Meniere’s disease according to the American Academy of Otolaryngology-Head and Neck Surgery (AAO-HNS) 1995 or delayed endolymphatic hydrops based on Schuknecht’s definition^1^,^4^.
4. They defined abnormal VEMPs (absent or decreased)^12^ as follows:

- When the latencies of c-VEMP, each peak (p13, n23) and amplitude (p13 to n23) were measured, the interaural amplitude difference ratio was greater than the mean of normal range plus 2 × standard deviation (SD);
- The peak-to-peak c-VEMP amplitude was absent or decreased;
- c-VEMP threshold shifts exceeded the mean of normal range plus 2×standard deviation (SD) (delayed response).
- Biphasic waveform of o-VEMP was absent after at least 50 responses;
- o-VEMP asymmetry ratio >40%.

Studies were excluded if:

1. They were reviews, editorial comments, case reports or letters;
2. They did not provide sufficient data;
3. They were animal studies.

### Data extraction

Two reviewers independently reviewed the eligible studies and extracted the relevant data, including first author, publication year, country of origin, race, study design, gender, mean age, clinical profiles, sample size, measurement protocol, and funding source. Moreover, they recorded the number of true-positive, false-positive, true-negative, and false-negative data for each study. Discrepancies, if any, were resolved by mutual discussion or the judgement of a third reviewer.

### Quality Assessment

The quality assessment of diagnostic accuracy studies (QUADAS) tool was used for quality evaluation. The QUADAS tool included 14 items, each being judged by yes, no, or unclear.

### Data analysis

If heterogeneity existed, a random-effects model was performed to examine the summary sensitivity, specificity, positive likelihood ratio, negative likelihood ratio, and diagnostic odds ratio (DOR).

Furthermore, the summary receiver operating characteristic (sROC) curve summarized each pair of sensitivity and specificity into a single measure of accuracy and the diagnostic odds ratio. SROC curve were delineated and the area of SROC (AUC) was calculated to evaluate the diagnostic accuracy and consistency of VEMPs in the context of a meta-analysis^15^,^16^,^17^.

To identify potential sources of heterogeneity, we performed subgroup analysis, including race, study design, type of controls, timing of tests, testing methods, stages of baseline disease and fund resources.

Publication bias was evaluated by using Deek’s funnel plots.

All of statistical analyses were conducted by employing MIDAS module for STATA, version 11.2 (Stata Corp, College Station, Texas) and Meta-Disc, Version 1.4 (Unit of Clinical Biostatics, the Ramóny Cajal Hospital, Madrid, Spain).

## Additional Information

**How to cite this article**: Zhang, S. *et al.* Diagnostic Value of Vestibular Evoked Myogenic Potentials in Endolymphatic Hydrops: A Meta-Analysis. *Sci. Rep.* **5**, 14951; doi: 10.1038/srep14951 (2015).

## Figures and Tables

**Table 1:** Characteristics of included eligible studies.

Author	Country	Study design	Case N	Control N	TP	FN	FP	TN	Funding
2003 Yi-Ho Young^18^	Taiwan	prospective	40	40	16	24	0	40	Government
2008 Seok Min Hong^19^	Korea	prospective	29	29	20	9	0	29	No funding
1999 catherine de waele^20^	France	retrospective	59	37	27	32	4	33	No funding
2012 CHI-HSUAN HUANG 1^21^	Taiwan	prospective	50	50	22	28	0	50	No funding
2012 CHI-HSUAN HUANG 2^21^	Taiwan	prospective	50	50	19	31	0	50	No funding
2011 Stephanie M Winters 1^22^	Netherlands	prospective	31	55	14	17	1	54	No funding
2011 Stephanie M Winters 2^22^	Netherlands	prospective	37	55	27	10	1	54	No funding
2008 Giuseppe Magliulo 1^23^	Italy	prospective	22	22	8	14	0	22	No funding
2008 Giuseppe Magliulo 2^23^	Italy	prospective	22	22	7	15	0	22	No funding
2008 Giuseppe Magliulo 3^23^	Italy	prospective	22	22	7	15	0	22	No funding
2006 Gu¨ zin Akkuzu^24^	Turkey	prospective	20	34	10	10	2	32	No funding
2005 Shih-Wei Kuo 1^25^	Taiwan	prospective	12	12	8	4	0	12	No funding
2005 Shih-Wei Kuo 2^25^	Taiwan	prospective	12	12	4	8	0	12	No funding
2012 M. Geraldine Zuniga 1^26^	USA	prospective	20	56	4	16	0	56	Government
2012 M. Geraldine Zuniga 2^26^	USA	prospective	20	56	10	10	2	54	Government
2012 M. Geraldine Zuniga 3^26^	USA	prospective	20	56	2	18	2	54	Government
2006 Ming-Yee Lin 1^27^	Netherlands	prospective	17	24	17	0	0	24	Government
2006 Ming-Yee Lin 2^27^	Netherlands	prospective	6	24	5	1	0	24	Government
2012 ANA PAULA SERRA^28^	Brazil	prospective	12	66	6	6	0	66	Government
2012 Jaswinder S. Sandhu^29^	UK	prospective	12	16	12	0	0	16	No funding
2012 HSUN-MO WANG^30^	Taiwan	retrospective	79	60	30	49	0	60	No funding
2011 TOSHIHISA MUROFUSHI 1^31^	Japan	prospective	20	14	11	9	0	14	No funding
2011 TOSHIHISA MUROFUSHI 2^31^	Japan	prospective	20	14	9	11	0	14	No funding
2002 Yi-Ho Young^32^	Taiwan	prospective	10	16	3	7	0	16	No funding
2007 V OSEI-LAH 1^33^	UK	prospective	11	36	2	9	0	36	Government
2007 V OSEI-LAH 2^33^	UK	prospective	9	36	5	4	0	36	Government
2007 V OSEI-LAH 3^33^	UK	prospective	20	36	5	15	0	36	Government
2013 Min-Beom Kim^34^	Korea	prospective	41	66	14	27	0	66	No funding
2013 Chuan-Yi Lin 1^35^	Taiwan	prospective	50	32	31	19	0	32	No funding
2013 Chuan-Yi Lin 2^35^	Taiwan	prospective	50	32	40	10	0	32	No funding
2006 CHUN-NAN CHEN^36^	Taiwan	prospective	14	14	10	4	0	14	Government
2009 T Murofushi^37^	Japan	prospective	11	16	5	6	0	16	No funding
2009 Chen-Han Chou1^38^	Taiwan	prospective	7	40	3	4	0	40	Government
2009 Chen-Han Chou2^38^	Taiwan	prospective	7	40	4	3	0	40	Government
2010 Naoya Egami 1^39^	Japan	retrospective	26	26	19	7	19	7	Government
2010 Naoya Egami 2^39^	Japan	retrospective	7	7	4	3	4	3	Government
2011 Chi-Hsuan Huang 1^40^	Taiwan	prospective	20	20	13	7	8	12	Government
2011 Chi-Hsuan Huang 2^40^	Taiwan	prospective	20	20	5	15	0	20	Government
2011 Chi-Hsuan Huang 3^40^	Taiwan	prospective	20	20	9	11	3	17	Government
2011 Chi-Hsuan Huang 4^40^	Taiwan	prospective	20	20	5	15	0	20	Government
2013 Naoya Egami 1^13^	Japan	prospective	114	94	57	57	22	72	Government
2013 Naoya Egami 2^13^	Japan	prospective	22	94	21	1	22	74	Government
2012 Mei-Chun Lin 1^41^	Taiwan	prospective	20	20	14	6	11	9	No funding
2012 Mei-Chun Lin 2^41^	Taiwan	prospective	20	20	9	11	6	14	No funding
2011 Rachael L. Taylor 1^42^	Australia	prospective	60	70	30	30	0	70	Government
2011 Rachael L. Taylor 2^42^	Australia	prospective	60	70	24	36	0	70	Government
2006 Timmer Ferdinand C A^43^	USA	retrospective	82	24	11	71	0	24	Government
2002 Yi-Ho Young^44^	Taiwan	prospective	20	20	11	9	0	20	Government
2009 Bernhard Baier^45^	Germany	prospective	16	126	11	5	0	126	No funding
2013 Young Joon Seo^46^	South Korea	prospective	26	26	25	1	0	26	No funding

**Table 2:** Subgroup analysis for accuracy of VEMP for MD detection.

Subgroup	N	Sensitivity	Specificity	DOR	AUC
Race					
Caucasian	12	0.43	0.99	57.25	0.95
Asian	18	0.53	0.92	28.59	0.69
Study design					
prospective	26	0.52	0.95	50.54	0.81
retrospective	4	0.36	0.94	10.62	0.78
Control					
health	20	0.49	0.96	44.93	0.82
patients	10	0.48	0.94	35.37	0.78
Attacks					
Yes	30	0.49	0.95	38.44	0.78
No	2	0.44	1.00	30.43	—
Methods					
Air conduct	29	0.48	0.95	36.48	0.76
Bone conduct	3	0.54	1.00	59.46	0.99
o-VEMP	10	0.48	0.96	20.96	0.45
c-VEMP	27	0.49	0.95	39.36	0.81
Click	6	0.45	0.95	22.18	0.59
Tone burst	23	0.48	0.99	60.66	0.97
Stage(c-VEMP)					
I	4	0.47	0.85	4.01	0.64
II	5	0.49	0.86	16.32	0.65
III	5	0.46	0.86	11.15	0.31
IV	4	0.54	0.85	12.13	0.39
Funding					
Government	13	0.45	0.94	27.60	0.77
No	17	0.52	0.97	53.63	0.80

DOR = Diagnostic Odds Ratio; AUC = the area under the summary receiver operating characteristic curve
